# C3 in *Pomacea canaliculata*: a conserved effector with developmental and immune roles

**DOI:** 10.3389/fcimb.2026.1782659

**Published:** 2026-06-30

**Authors:** Filippo Bertolasi, Sandro Sacchi, Alessandro Vezzi, Gabriele Sales, Anita Ferri, Davide Malagoli, Nicola Franchi

**Affiliations:** 1 Department of Life Sciences, University of Modena and Reggio Emilia, Modena, Italy; 2 Department of Biology, University of Padova, Padova, Italy; 3 National Biodiversity Future Center (NBFC), Palermo, Italy

**Keywords:** C3, cell clearance, complement evolution, complement system, development, immunity, invertebrate

## Abstract

C3 is the central effector of the complement system and one of the most conserved molecules of metazoan innate immunity. Despite its relevance, little is known about complement components in Lophotrochozoa. Here, we characterise the C3 ortholog of the freshwater gastropod *Pomacea canaliculata* (PcC3) using long-read transcriptomics, phylogenetics, expression profiling and immunohistochemistry. PcC3 displays a fully conserved domain architecture and clusters phylogenetically in agreement with metazoan relationships. Following LPS challenge, PcC3 is up-regulated in haemocytes and unexpectedly in the ampulla, but not in the posterior kidney, revealing functional specialization of immune tissues. Early developmental transcriptomes reveal strong and sustained PcC3 activation from 3 to 9 dpf. These data suggest that PcC3 retains canonical immune functions but may also contribute to developmental and homeostatic cell clearance, supporting *P. canaliculata* as a valuable model for complement biology in molluscs.

## 1 Introduction

The complement system represents a fundamental and ancient component of metazoan humoral innate immunity, acting as a first line of defence that promotes local inflammatory reactions and coordinates, in Gnathostomes, adaptive immune responses. In vertebrates this system is composed of a finely regulated network of plasma proteins, cell surface receptors, and regulators, comprising over 30 secreted or membrane-bound proteins (Sunyer et al., 1997; Pinto et al., 2003; Ricklin et al., 2016a).

In all metazoans studied to date, the activation pathways, whether classical, lectin, or alternative, converge on the activation of the central protein C3 (Peng et al., 2016; Sun et al., 2023). C3 is pivotal to the complement system, playing essential roles in immune defence, immune regulation, and immune pathology (Peng et al., 2016). It serves as the nexus of multiple activation pathways, driven by a positive feedback loop of C3b, and also acts as a crucial link between innate and acquired immunity (Meng et al., 2012; Peng et al., 2016; Ricklin et al., 2016b).

The third component of complement (C3) is recognized as the central protein and a functional hub of the entire system, serving as the convergence point for activation pathways and fuelling the amplification process of the immune response (Ricklin et al., 2016b).

Its active fragments, C3a and C3b, are vital for phagocytosis (Peronato et al., 2020a; Sun et al., 2023; Guo et al., 2025), respiratory burst (Ricklin et al., 2016b; Peronato et al., 2020a), and inflammatory processes (Ricklin et al., 2016b; Peronato et al., 2020c), with the anaphilotoxin C3a showing strong chemotactic activity in inflammatory responses (Ricklin et al., 2016b; Peronato et al., 2020c) and C3b serving an opsonic role (Peronato et al., 2020a; Sun et al., 2023; Guo et al., 2025).

Beyond its traditional role in pathogen clearance (Ricklin et al., 2016a, b), C3 is involved in a variety of homeostatic processes (Dodds, 2002; Rahman et al., 2020; Pittaluga et al., 2025). Notably, complement is engaged during synaptic pruning, a process mediated by powerful iC3b-CR3 mechanisms (Gurol et al., 2016; Vorup-Jensen and Jensen, 2018; Guo et al., 2025). This involvement has potential implications for the development of schizophrenia (Gurol et al., 2016; Woo et al., 2020). Furthermore, C3’s functions extend to tissue regeneration (Ricklin et al., 2016b; Bergamini et al., 2023; Mastellos et al., 2023; Accorsi et al., 2025), the clearance of cellular debris (Mastellos et al., 2023), the control of tumour cell progression (Macor and Tedesco, 2007; Sayegh et al., 2014) and retinal regeneration (Fernández-Sánchez et al., 2025).

Its importance is also underscored by its high evolutionary conservation, being a very ancient component that precedes the divergence between deuterostomes and protostomes (Meng et al., 2012; Wang et al., 2013; Elvington et al., 2016; Peng et al., 2016). C3 homologs have been identified in evolutionarily distant species, from the most primitive invertebrates, such as Cnidarians (Poole et al., 2016), up to Tunicates (Franchi and Ballarin, 2014; Franchi and Loriano, 2017; Peronato et al., 2020b, a, c; Peronato et al., 2021; Ballarin et al., 2024) and Vertebrates. This phylogenetic conservation demonstrates that C3 has maintained its function as an immune sentinel, playing a primary role in “non-self” recognition within a primordial phagocytic system (Gorbushin, 2018).

The evolution of the complement system in invertebrates, particularly within the Lophotrochozoa lineage which includes molluscs, remains a less explored area (Gorbushin, 2018). Molluscs are economically, environmentally, and publicly significant organisms, and research into their stress responses provides valuable contributions to parasitology, conservation biology, and human welfare (Accorsi et al., 2013; Sacchi et al., 2024).

In molluscs, numerous C3 homologs have been identified in various species. These include *Crassostrea gigas* (Wang et al., 2017), *Mytilus coruscus* (Chen et al., 2018), *Sinonovacula constricta* (Peng et al., 2016) and *Littorina littorea* (Gorbushin, 2018). These mollusc C3s exhibit conserved structural domains, such as the alpha-2 macroglobulin (A2M) domain and the thioester site (Peng et al., 2016; Gorbushin, 2018). Unlike mammals, where C3 is primarily synthesized in the liver and macrophages (Marino et al., 2002; Kristensen et al., 2025), in molluscs it is expressed in various tissues, including haemocytes, gills, hepatopancreas, and mantle (Peng et al., 2016; Sun et al., 2023). C3 expression in molluscs is induced in response to bacterial stimuli and PAMPs (Pathogen-Associated Molecular Patterns) (Peng et al., 2016; Sun et al., 2023). Functionally, mollusc C3 is involved in promoting haemocyte phagocytosis (Gorbushin, 2018; Guo et al., 2025), inhibiting bacterial growth and promoting pathogen degradation (Guo et al., 2025).

In this context, the freshwater gastropod *Pomacea canaliculata*, also known as the golden apple snail, has emerged as an exceptional study model in various research areas, including ecophysiology (Accorsi et al., 2013), parasitology (Boraldi et al., 2021), regeneration biology (Accorsi et al., 2025), comparative and developmental immunology (Sacchi et al., 2024). This invasive species, originally from South America (Cueto et al., 2015) and now widely distributed in Asia (Li et al., 2025), exhibits remarkable resistance to pollutants and hypoxia (Sacchi et al., 2024), and is an intermediate host for the zoonotic nematode *Angiostrongylus cantonensis*, the etiological agent of human eosinophilic encephalitis (Boraldi et al., 2021). Despite limited previous information on its specific immune system, *P. canaliculata* possesses an intrinsically aggressive and reactive immune system, capable of effectively combating bacterial infections and parasitism (Accorsi et al., 2013, 2014). Its circulating haemocytes have been well-characterized, distinguishing into two main populations: Group I (small, with prohemocyte characteristics) and Group II (larger, with agranular or granular cytoplasm and capable of phagocytosis) (Accorsi et al., 2013, 2014). These haemocytes demonstrate considerable rapidity in modifying their morphological characteristics *ex vivo*, confirming their high reactivity (Accorsi et al., 2013). Furthermore, *P. canaliculata* shows an extraordinary capacity for regeneration in adults, being able to entirely reconstruct its camera-type eye (Accorsi et al., 2025) and cephalic tentacles (Bergamini et al., 2023). Haemocytes are present in the forming blastema during the early stages of regeneration (Bergamini et al., 2023; Accorsi et al., 2025) suggesting their active role not only as scavenger cells for removing cellular debris, but also in the regrowth of neural and other tissues (Bergamini et al., 2021). The combination of a dynamic immune system and remarkable regenerative capabilities makes *Pomacea canaliculata* a promising model for future studies on mollusc immunity and developmental and regenerative biology (Accorsi et al., 2013, 2014, 2025; Boraldi et al., 2019; Bergamini et al., 2021).

## 2 Materials and methods

### 2.1 Whole transcriptome samples retrieval and expression analysis

A total of 85 RNA-seq libraries of *Pomacea canaliculata* were retrieved from the NCBI Short Read Archive (SRA) using the SRA Toolkit (v2.11.0) fasterq-dump command. Raw FASTQ file quality was initially assessed using fastp (v0.20.1) to generate per-sample quality control reports. Adapter sequences and low-quality regions were removed using Trimmomatic (v0.39) in paired-end mode with the following parameters: Illuminaclip: TruSeq3-SE:2:30:10, slidingwindow:4:20, and minlen:50. Cleaned reads were re-evaluated with fastp to confirm sequence integrity. The sequences were then indexed against a curated set of target sequences, including PcC3, for *in silico* expression quantification using Salmon (v1.8.0) in quasi-mapping mode with default settings. Each library was quantified individually, and the resulting per-sample quantification outputs were then aggregated into two expression matrices corresponding to days post-fertilization (dpf) samples and adult haemocyte samples for downstream statistical analysis.

### 2.2 Dissection, slide preparation, histology, and immunohistochemistry

Selected snails were anesthetized on ice for 20 min prior to dissection to inhibit muscular and defensive responses and minimize animal distress. Posterior kidneys were excised from five adult individuals and immediately immersed in either fresh Bouin’s solution or 4% paraformaldehyde (PFA) (Sigma-Aldrich Corporation, Missouri, USA). Bouin’s fixative was prepared by mixing 15 mL picric acid, 5 mL 40% formaldehyde and 1 mL glacial acetic acid, then filtering through filter paper.

Tissues were dehydrated the day after fixation by sequential immersion in graded ethanol: samples were transferred from fixative into 50% ethanol in distilled water for 1 h 30 min, then into 70% ethanol for 1 h 30 min, followed by 90% and 100% ethanol (half‐volume fill) each for 1 h 30 min. After 100% ethanol, tubes were filled to volume with xylene for 1 h 30 min, emptied, refilled halfway with fresh xylene, and incubated at 60 °C for 1 h 30 min. Tubes were then brought to volume in molten paraffin at 60 °C for 1 h, emptied, and refilled with pure paraffin for 2 h with the cap removed to ensure complete xylene evaporation. The paraffin–tissue mixture was gently poured into glycerol-coated metal embedding moulds and cooled overnight at 4–6 °C. Meanwhile, histological slides were degreased with acetone, labelled, and prepared for sectioning.

The paraffin‐embedded tissue was sectioned at 7 µm using a rotary Microm HM310 microtome (Thermo Fisher Scientific, Walldorf, Germany). Sections were floated onto water‐coated slides, warmed on a 34–36 °C hot plate, and allowed to air‐dry overnight; the next morning, slides were ready for immunolocalization or staining.

Slides were deparaffinized and rehydrated, then permeabilized in PBS with 0.1% Tween-20 for 30 min and washed in PBS (3 × 5 min). To block non-specific binding, sections were incubated for 30 min in 2% blocking reagent in PBS in a humid chamber, then, except for negative controls, incubated overnight at 4 °C with 500 µL 1:500 rabbit polyclonal anti-PcC3 antibody (custom made by Bio-Fab research srl, Roma, Italy) in PBS (Rb2707). Slides were then incubated for 1 h at room temperature with 500 µL biotinylated goat anti-rabbit IgG (1:200), during which the avidin–biotin–peroxidase complex was prepared and subsequently applied for 30 min. Immunolabeling was detected with 0.5 mg/mL DAB in Tris–HCl containing 0.01% H_2_O_2_. Finally, slides were dehydrated, mounted with Eukitt resin, and air-dried before observation.

Haemocytes from 8 adult specimen of *Pomacea canaliculata* were fixed in Bouin’s solution as described for the immunohistochemistry, permeabilized in PBS containing 0.05% Tween-20 for 10 min, and then blocked with 2% blocking reagent (in PBS) for 30 min at room temperature. For each of the eight animals tested, haemocytes were collected and subsequently divided into two aliquots: one exposed to 1 mg/ml LPS for 1 h and the other maintained as an untreated control in Snail solution (51.3 mM NaCl, 1.7 mM KCl, 4.1 mM CaCl_2_·2H_2_O, 1.5 mM MgCl_2_·6H_2_O, 2 mM NaHCO_3_, 10 mM glucose e 10 mM HEPES pH 7.5). The subsequent steps of the protocol strictly followed those previously described. The percentage of immune positive cells was estimated by counting the haemocytes (at least 200 cells per slide), in 10 optic fields per slide at a magnification of 700 x. Statistical significance was assessed through paired t-test on the mean percentages of the eight biological replicates. Results are expressed as mean ± SD, and a p-value< 0.05 was considered statistically significant.

Untreated tissue slides were used as histological references to localize signals within tissue sections. All staining procedures included standard rehydration and dehydration steps using graded alcohols and xylene, followed by mounting with Eukitt as previously described.

The polyclonal antibody specific against PcC3 was produced in rabbit by Bio-Fab Research (Rome, Italy) and, following purification, was tested by ELISA using serial dilutions. All immunological analyses were repeated on tissues collected from five different animals, and the results were consistent across biological replicates.

### 2.3 Oxford nanopore library preparation and sequencing

cDNA libraries from haemocytes and posterior-kidney were prepared using the cDNA-PCR Sequencing Kit (SQK-PCS111, Oxford Nanopore Technologies), following the PCS_9143_v111_revI_01Dec2021-minion protocol. For each library, 200 ng of total RNA were used as input material. Following reverse transcription and strand-switching, 20 μl of each reverse-transcribed sample were used to set up four 50 μl PCR reactions to select full-length transcripts. PCR reaction conditions were set as follows: initial denaturation, 95 °C 30 s, followed by 14 cycles of denaturation 95 °C 15 s, annealing 62 °C 15 s, extension 65 °C 3 min, and a final extension 65 °C 6 min. AMPure XP beads (Beckman Coulter, CA, USA) were used for amplified-cDNA purification according to the protocol. The obtained libraries were analysed for DNA size and quality, using the D5000 DNA ScreenTape Assay of the Agilent TapeStation (Agilent, CA, USA), and quantity, using the Qubit™ DNA High Sensitivity kit (Thermo Fisher Scientific, MA, USA). A total of 25 fmol of each library were used for adapter ligation. Flow cell (FLO-MIN106) checking, priming and loading were carried out according to the manufacturer’s instructions. Sequencing was performed using the MinION Mk1C technology (Oxford Nanopore Technologies, Oxford, UK) with an initial bias voltage of −180 mV. Samples were sequenced for a total runtime of 64 h.

### 2.4 Transcriptome assembly

Raw signal data generated by the Oxford Nanopore sequencer were base−called with Dorado (v0.6.2) using the high−accuracy (HAC) model. This produced 20,729,714 reads for the haemocyte library and 25,059,799 reads for the posterior−kidney library. Full−length reads were identified, oriented, and trimmed with Pychopper (v2.7.9). The tool flagged 1,106,133 haemocyte reads (5.3 % of total) and 1,433,130 kidney reads (5.7 %) as unusable; these reads were excluded from further analysis.

The filtered reads were then assembled into transcripts with RNA−Bloom (v2.0.1), applying the “long” preset specifically designed for ONT data. The assembly generated a total of 197,574 transcripts for the haemocyte dataset and 154,051 transcripts for the kidney dataset.

### 2.5 Analysis of PcC3 gene copy number

To determine the copy number of the *C3* gene in *P. canaliculata*, we used a multi-level bioinformatic approach. First, the known *PcC3* transcript sequence was mapped against the reference genome using minimap2 (v2.26-r1175) with the -ax splice preset to account for introns and splice junctions. Genomic coordinates of the alignments were consolidated using bedtools (v2.31.1) to define the locus boundaries. To exclude potential assembly artifacts, mapping depth was calculated using samtools (v1.19.2) and normalized against the average depth of the chromosome.

Furthermore, to ensure no unannotated copies were missed, a TBLASTN search was performed against the entire whole-genome assembly using NCBI BLAST+ (v2.12.0) with the PcC3 protein sequence as a query. For protein-level validation, a BLASTP search was conducted against the official *P. canaliculata* proteome. All homology searches were filtered using an E-value threshold of $1\times 10^{-5}$.

### 2.6 Primer design, RNA extraction, cDNA synthesis, cloning and sequencing

Total RNA was extracted from each tissue sample using the Quick-RNA MiniPrep kit (Zymo Research, California, USA) according to the manufacturer’s instructions. RNA yield and purity were determined by measuring A_260_/_280_ and A_260_/_230_ ratios on a NanoDrop One spectrophotometer (Thermo Fisher Scientific, Massachusetts, USA), and RNA integrity was confirmed by electrophoresis on a 1.5% agarose gel stained with SYBR Safe DNA gel stain (Invitrogen, California, USA).

For each reaction, 1 µg of total RNA was combined with 0.5 µL of random primers (provided) and RNase-free water to 5 µL, then incubated at 70 °C for 5 min. Reverse transcription mix (10 µL) containing 1.25 µL dNTPs (10 mM each), 5 µL M-MLV 5X Reaction Buffer (Promega), 1 µL RNase Inhibitor (25 U), 1 µL MMLV Reverse Transcriptase (200 U)(Promega), and RNase-free water to a final volume of 15 µL was added to each primer-RNA mixture. Reactions were incubated at 37 °C for 60 min and then held at 4 °C before downstream applications.

PCR amplification of cDNA was performed in 25 µL reactions using the GoTaq Pro G2 DNA Polymerase Kit (Promega, Wisconsin, USA). Each reaction contained 100 ng of reverse-transcribed cDNA, 5 µL of 10× GoTaq Reaction Buffer, 0.5 µL of 10 mM dNTP mix (Promega), 0.5 µL of each forward and reverse primer (10 µM; sequences listed in **Supplementary Table 1**), 0.2 µL of GoTaq DNA Polymerase (5 U/µL) and RNase-free water to a final volume of 25 µL. Thermal cycling was carried out on an Applied Biosystems (California, USA) 2720 Thermal Cycler with an initial denaturation at 95 °C for 5 min, followed by 40 cycles of 95 °C for 30 s, 56 °C for 30 s and 72 °C for 40 s to 1 min, and a final extension at 72 °C for 10 min. PCR products were resolved on 1.5% agarose gels containing 0.5 µg/mL ethidium bromide (Sigma-Aldrich) in 1× TAE buffer at 70 V for 60 min and visualized under UV illumination using a Gel Doc XR system (Bio-Rad).

The sequencing was performed by Eurofins Genomics (Ebersberg, Germany).

### 2.7 Transcriptional expression analysis by real-time PCR

For the analysis of PcC3 transcript expression, five (n) animals were injected into the hemolymphatic sinus of the muscular foot with 100 µl of an LPS solution (1 mg/ml in Snail solution), while five control animals received 100 µl of Snail solution only. One hour after treatment, the animals were kept on ice for 40 minutes and subsequently dissected to collect the organs of interest.

Real‐time semiquantitative PCR was carried out using PcEF1-alpha as housekeeping on a CFX Duet Real-Time PCR System (Bio-Rad). Gene-specific primers for *PcC3* and P*cEF1-alpha* were designed and synthesized by a commercial provider (Eurofins Genomics, Ebersberg, Germania); primer stocks were prepared at 3 µM, and 0.5 µL of each primer (forward and reverse) was included per reaction. Each 10 µL reaction comprised 4 µL of cDNA (diluted 1:5 in RNase-free water) and 5 µL of SsoAdvanced Universal SYBR Green Supermix (Bio-Rad, California, USA). Thermal cycling conditions consisted of an initial denaturation at 95 °C for 2 min, followed by 40 cycles of denaturation at 95 °C for 5 s and annealing/extension at 60 °C for 30 s. Upon completion of amplification, a dissociation curve was generated by heating to 95 °C for 5 s, cooling to 65 °C for 5 s and reheating to 95 °C for 5 s to verify product specificity. Threshold cycle (Ct) values were determined using CFX Maestro software (Bio-Rad, California, USA).

### 2.8 Phylogenetic analysis

Orthologous C3 protein sequences were retrieved from the NCBI and UniProt databases and compiled into a single FASTA file. Multiple sequence alignment was performed using MUSCLE with default settings within the MEGA (v. 11) software. Model selection was performed in MEGA by testing multiple amino acid substitution models. The *Le and Gascuel* (2008) model with gamma-distributed rates (LG+G, parameter = 3.1125) was identified as the best-fitting model based on likelihood criteria (Log Likelihood: -119857.38) and was therefore selected for phylogenetic reconstruction. A gamma distribution with four discrete categories was applied. Maximum-likelihood analysis was conducted starting from an initial tree generated by the neighbour-joining algorithm. The tree search employed the nearest neighbour interchange (NNI) heuristic to explore alternative topologies. Node support was assessed via 1000 bootstrap replicates. An outgroup composed of alpha-2 macroglobulin (A2M) sequences from multiple taxa was included to root the phylogenetic tree.

### 2.9 Statistical analysis

To allow for comparison across different sequencing libraries, raw transcript counts (NumReads) obtained from Salmon were normalized as Counts Per Million (CPM). Specifically, the scaling factor was calculated based on the total sum of the curated target transcripts. For all downstream statistical analyses, these values were log_2_-transformed (log_2_-CPM) to stabilize the variance and to reduce the skewness of the data distribution.

Developmental data (dpf samples) were sourced from a single BioProject (PRJNA473253, Stowers Institute for Medical Research), providing a controlled time-course with uniform experimental conditions. To compare PcC3 expression pairwise across different post-fertilization developmental stages, a one-way ANOVA was performed to assess the presence of overall differences among stages, followed by Tukey’s *post-hoc* test for multiple pairwise comparisons. For the adult haemocyte samples, retrieved from a single study (PRJNA476647, Institute of Zoology, Chinese Academy of Sciences), transcript abundance was compared between sexes using Welch’s t-test. In all cases, a normal distribution was assumed following log_2_-transformation, and results were considered statistically significant at p< 0.05.

Real-time PCR results were analysed using a two-way ANOVA to evaluate the effect of tissue type, LPS treatment and their interaction. For multiple comparisons, Tukey’s HSD *post-hoc* test was applied, and significant differences (p< 0.05) were represented using a compact letter display (CLD). Comparisons between groups in the cell labelling experiments were performed using an unpaired t-test.

## 3 Results

### 3.1 Gene, transcript and protein organization

Full-length transcriptomes generated through Oxford Nanopore sequencing showed high overall completeness. The completeness of the transcriptome assemblies was assessed using BUSCO, searching against a set of 672 conserved orthologous groups. For the haemocyte sample, the analysis revealed a high level of completeness, identifying 93.2% of the expected conserved genes (626/672). Only a marginal fraction of genes were classified as fragmented (4.2%) or missing (2.7%). For the posterior kidney samples, a completeness score of 82.0% was obtained, with 551 BUSCOs identified as complete. In this case, 10.4% of the genes were classified as fragmented and 7.6% as missing. Overall, these results indicate that both assemblies provide a robust and largely complete representation of the expressed gene repertoire, with higher completeness observed in haemocytes compared to posterior kidney tissue.

The PcC3 transcript is 6718 bp in length and contains a coding sequence (CDS) of 5559 bp, with 5’ UTR regions of 210 bp and 3’ UTR regions of 978 bp. The gene structure was analysed by comparing the cDNA to genomic sequences. *PcC3* includes 39 exons, with a start codon ATG localized in the first exon and the TGA stop codon in the last exon. Notably, despite the high sensitivity of long-read sequencing, only one dominant transcript was observed.

In silico translation identified a protein of 1852 amino acids. Domain prediction revealed the presence of a signal peptide (residues 1–21), followed by two A2M domains (alpha-2 macroglobulin; residues 474–624 and 790–880, respectively). The protein sequence also reveals a conserved C3-convertase cleavage site, corresponding to the canonical tetrabasic (“tetra R”) motif located approximately between residues 710 and 720, consistent with previously described molluscan C3 sequences (Gorbushin, 2018). Sequence inspection of PcC3 revealed the presence of two putative proteolytic cleavage sites compatible with iC3b-type processing. The first site is located approximately at position 1440 of the PcC3 amino acid sequence, while the second is located around position 1455. In both cases, the motifs include basic residues (Lys, K) in positions corresponding to the canonical Factor I cleavage regions described in vertebrate C3. Although Arg (R) is typically reported at these positions in mammals, the presence of Lys maintains the basic character of the P1 residue, consistent with cleavage specificity of trypsin-like serine proteases. These motifs are positioned within the C-terminal region of the α-chain, consistent with the location of regulatory processing sites in other metazoan C3 molecules. Additional cleavage sites leading to C3dg and C3d fragments, as described in vertebrates, were not clearly identifiable.

A thiol-ester domain (alpha-2 macroglobulin thiol-ester bond-forming region) is located between residues 1029 and 1058. The catalytic histidine residue, required for thioester-mediated target binding upon activation, is conserved and located at the expected functional position downstream of the thioester site (around residue 1130).

**Figure 1:**
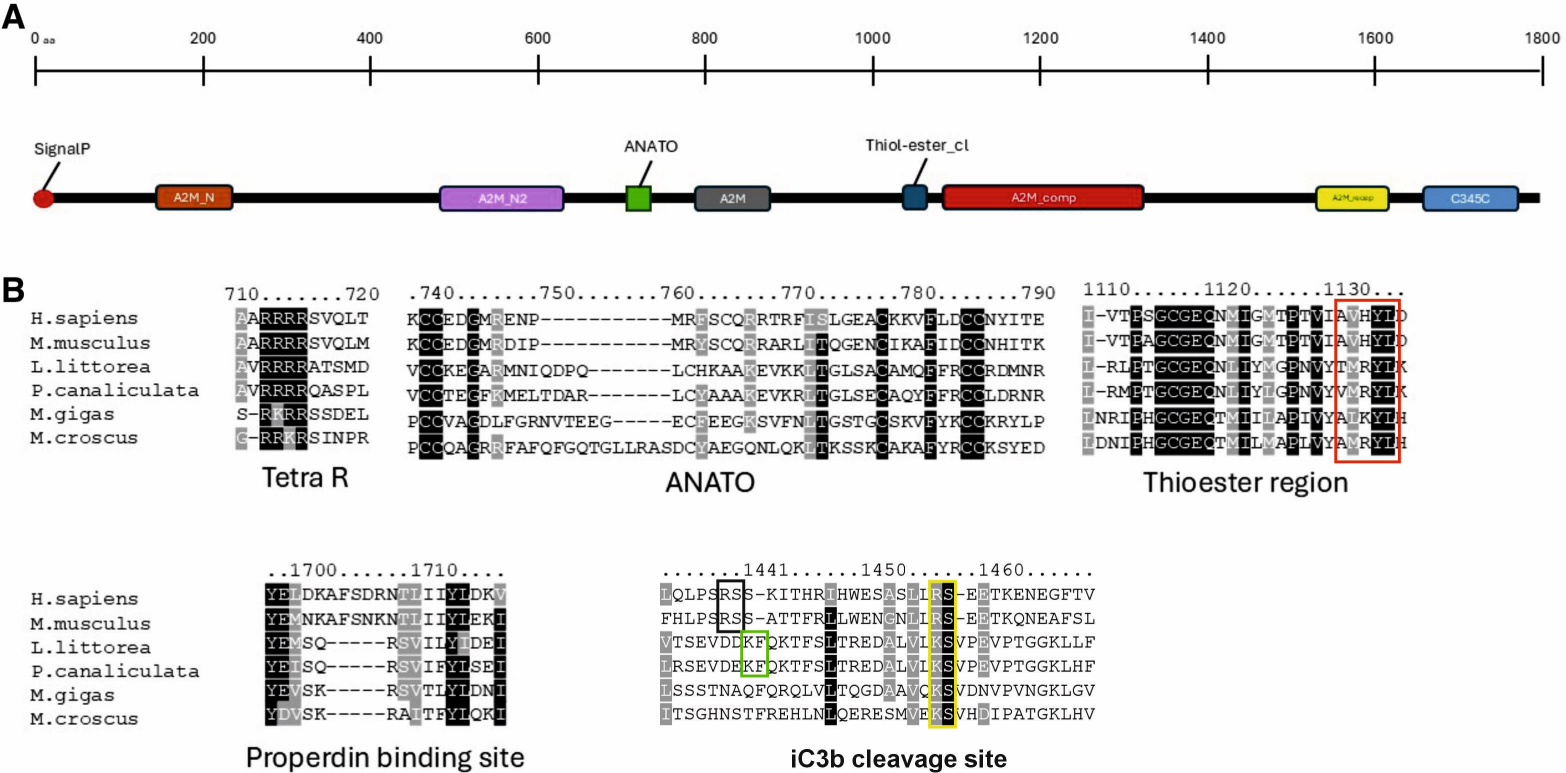
**(A)** Schematic domain organization of C3 protein in *Pomacea canaliculata*. a2-M N: N terminal alpha-macroglobulin domain; a2-M N: N terminal alpha-macroglobulin domain 2; ANATO: anaphylatoxin homologous domain; a2-M M: median alpha-macroglobulin domain; a2-M complement: alpha-macroglobulin complement component; a2-M rece: alpha-macroglobulin receptor; NTR/C345C: NTR/C345C domain. Numbers refer to the length of the amino acid sequences. **(B)** Alignment of P. canaliculata PcC3 functional regions with *Homo sapiens*, *Mus Musculus*, *Littorina littorea*, *Magellana gigas (Crassostrea gigas)* and *Mytilus croscus*. Aminoacid numbers refers to PcC3 sequence. The red box indicates the catalytic histidine residue that mediates target binding via the thioester site. The black box indicates the conserved mammalian P1 site for iC3b generation. The green box indicates the putative P1 site in Pomacea canaliculata and Littorina littorea. The purple box indicates the conserved P2 site involved in iC3b generation by Factor I (or its orthologues).

The protein structure is completed by an A2M receptor domain (A-macroglobulin receptor domain; residues 1540–1628) and a C345C domain (Netrin C-terminal domain; residues 1671–1786) (**Figure 1**).

### 3.2 Genomic evidence for a single *C3* locus in *P. canaliculata*

The genomic survey unequivocally identified a single *C3* locus on Chromosome 8, spanning approximately 23.5 kb (Chr8: 3,988,526–4,012,004). The structural integrity of this locus was supported by a uniform, normalized mapping depth; the coverage profile remained strictly consistent with the chromosomal average (1:1 ratio), ruling out the presence of tandem duplications or high-identity paralogs collapsed during the assembly process.

Homology-based searches provided further confirmation of this unique architecture at both the nucleotide and protein levels. BLASTN analysis against the genome assembly identified a single significant hit on Chromosome 8 with an E-value of 0.0 and 97.48% identity (1237/1269 bp). Consistently, TBLASTN searches against the whole-genome assembly and BLASTP searches against the proteome identified only one high-identity match on Chromosome 8 (TBLASTN E-value: 2e-46; BLASTP identity: ~99%, ID: Pcan|008G000232.mRNA.1). While secondary signals were detected on Chromosomes 2, 12, and 13, these exhibited significantly lower sequence identity (<32–40%). Collectively, these data demonstrate that *P. canaliculata* possesses a single-copy *C3* gene, distinguishing it from other molluscan lineages that have undergone extensive complement system expansions.

### 3.3 Differential PcC3 transcriptional response to LPS challenge

**Figure 2:**
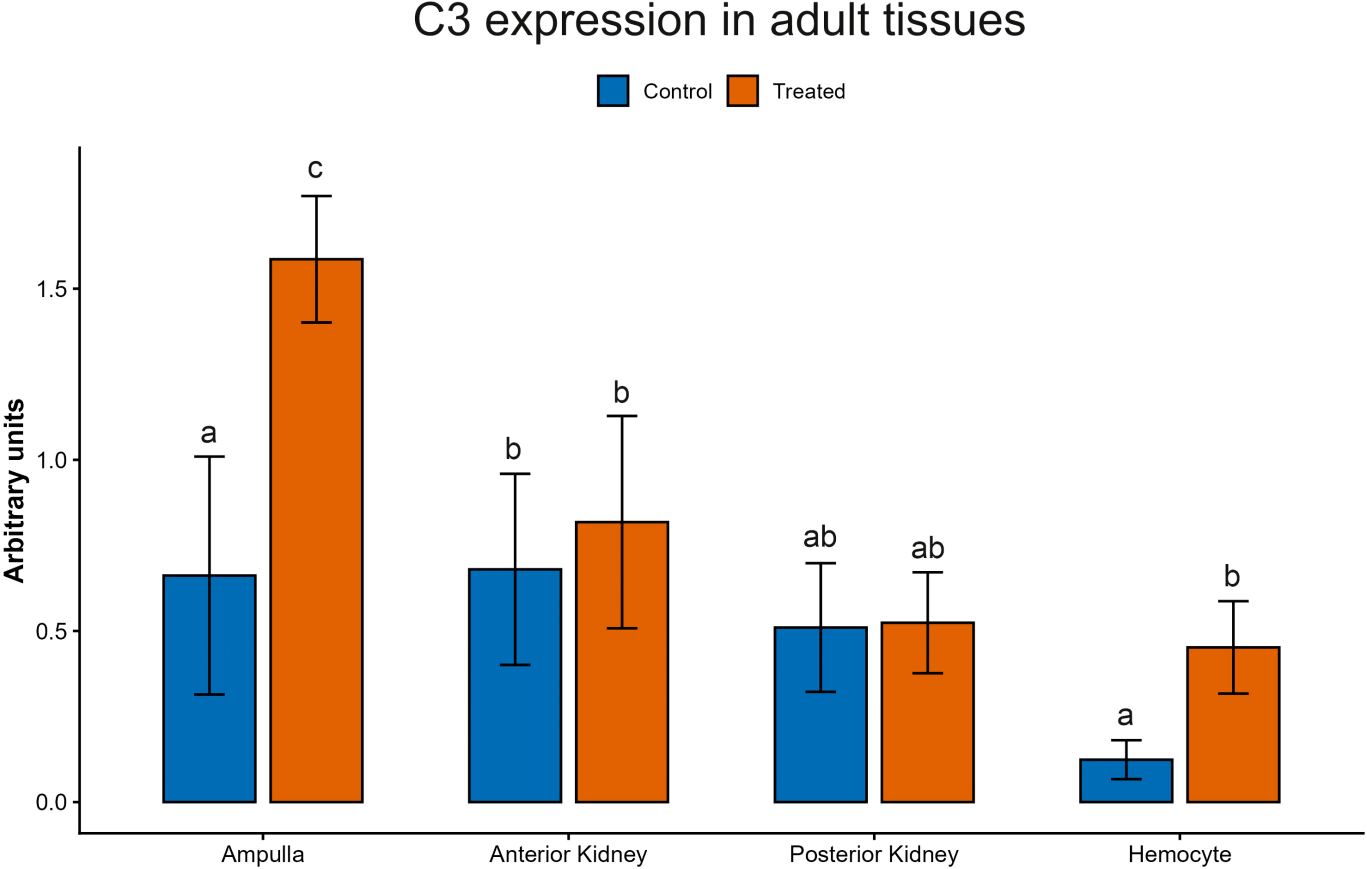
qPCR analysis of *Pomacea canaliculata* C3 (PcC3) expression in adult tissues under control and LPS-treated conditions. Expression levels were normalized to the endogenous housekeeping gene Elongation Factor 1 alpha (EF1-alpha). Data are presented as mean ± SD (n=5 biological replicates). Bars labelled with different letters differs significantly (two-way ANOVA p<0.05).

The analysis of PcC3 transcript expression revealed a tissue-specific response to LPS challenge (Two Way ANOVA, interaction p = 0.0004) (**Figure 2**). The lowest basal expression was observed in control haemocytes (group “a”). Notably, following LPS treatment, haemocytes showed a substantial upregulation shifting to group “b”.

The most statistically significant upregulation occurred in the ampulla (from group “b” to “c”). In contrast, the anterior and posterior kidneys remained transcriptionally stable, without significant differences observed between control and treated groups (groups “b” and “ab”).

### 3.4 PcC3 developmental and sex-specific expression

**Figure 3:**
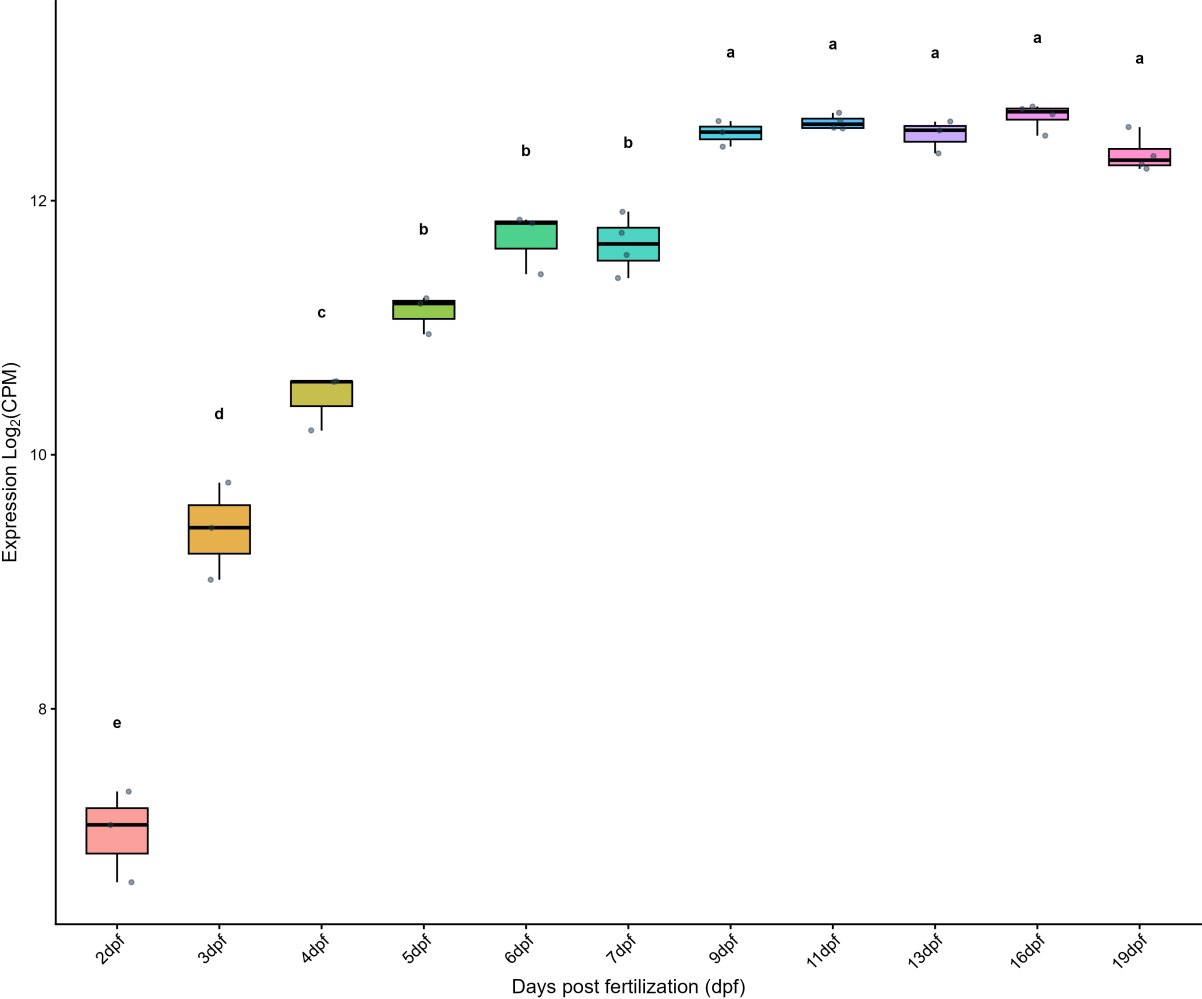
In silico expression analysis of *Pomacea canaliculata* C3 (PcC3) during the early development, expressed as Log_2_ CPM (Counts Per Million). Expression profile during early developmental stages (days post-fertilization and hatching). Different letters indicate statistically significant differences among developmental stages (p = 0.05). Stages sharing at least one letter are not significantly different. Letters follow the compact letter display (cld) convention: groups sharing at least one letter are not significantly different, whereas groups with different letters differ significantly (p< 0.05).

**Figure 4:**
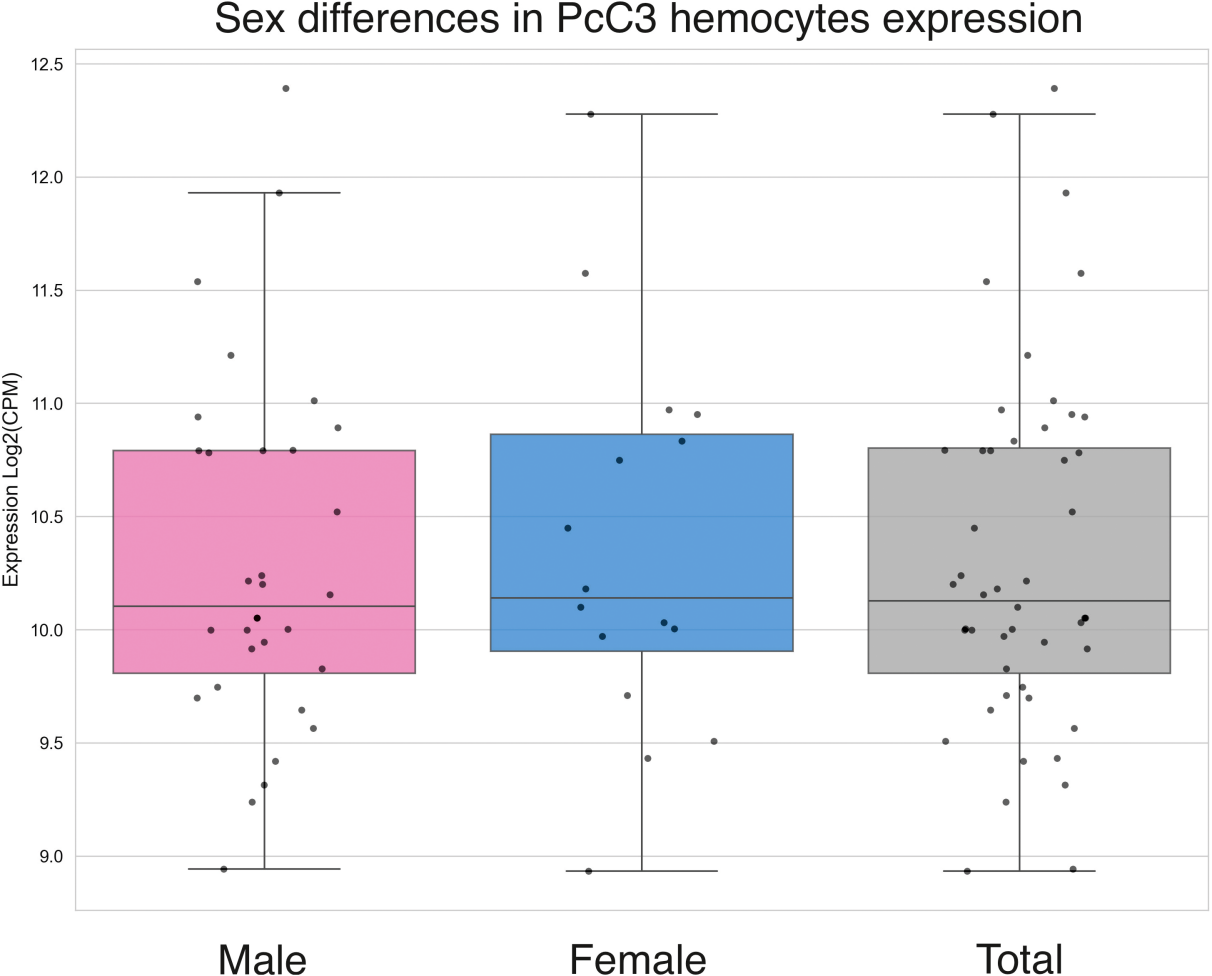
Expression levels of *PcC3* in adult haemocytes from different sexes.

Gene expression analysis (log_2_CPM) across various days post-fertilization (dpf) (**Figure 3**) revealed a temporal pattern. Expression showed a significant increase from the early stages (2–4 dpf), spiking between 6 and 9 dpf, and then establishing a stable plateau that persisted until 19 dpf. To evaluate potential sex-specific differences, transcriptomic haemocytes data from various male and female specimens were retrieved from the SRA archive (**Supplementary Table 2**) and analysed considering gene expression (log_2_CPM). The analysis showed no significant differences between the sexes (**Figure 4**).

### 3.5 Cellular localization of PcC3 in circulating haemocytes and posterior kidney

**Figure 5:**
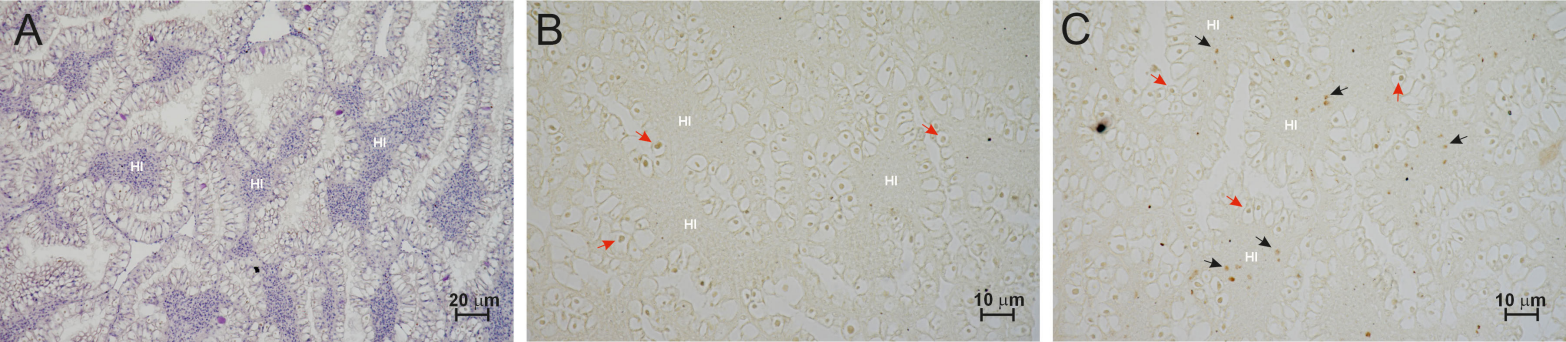
Immunohistochemical localization of PcC3 in the posterior kidney of *Pomacea canaliculata*. **(A)** Haematoxylin-eosin stained posterior kidney section. **(B)** Immunohistochemistry on posterior kidney negative control (no primary antibody). **(C)** Immunoistochemistry with primary antibody. Cells labelled by the antibody are indicated by black arrows; pigmented granules are indicated by red arrows. HI denotes haemocyte islets.

The posterior kidney was observed histologically in adult to characterize its morphology and observe haemocyte islets (**Figure 5**).

The results of the immunohistochemistry technique with anti PcC3 antibody in the posterior kidney reveals evident staining in haemocytes within hemocyte islets (**Figure 5C**).

**Figure 6:**
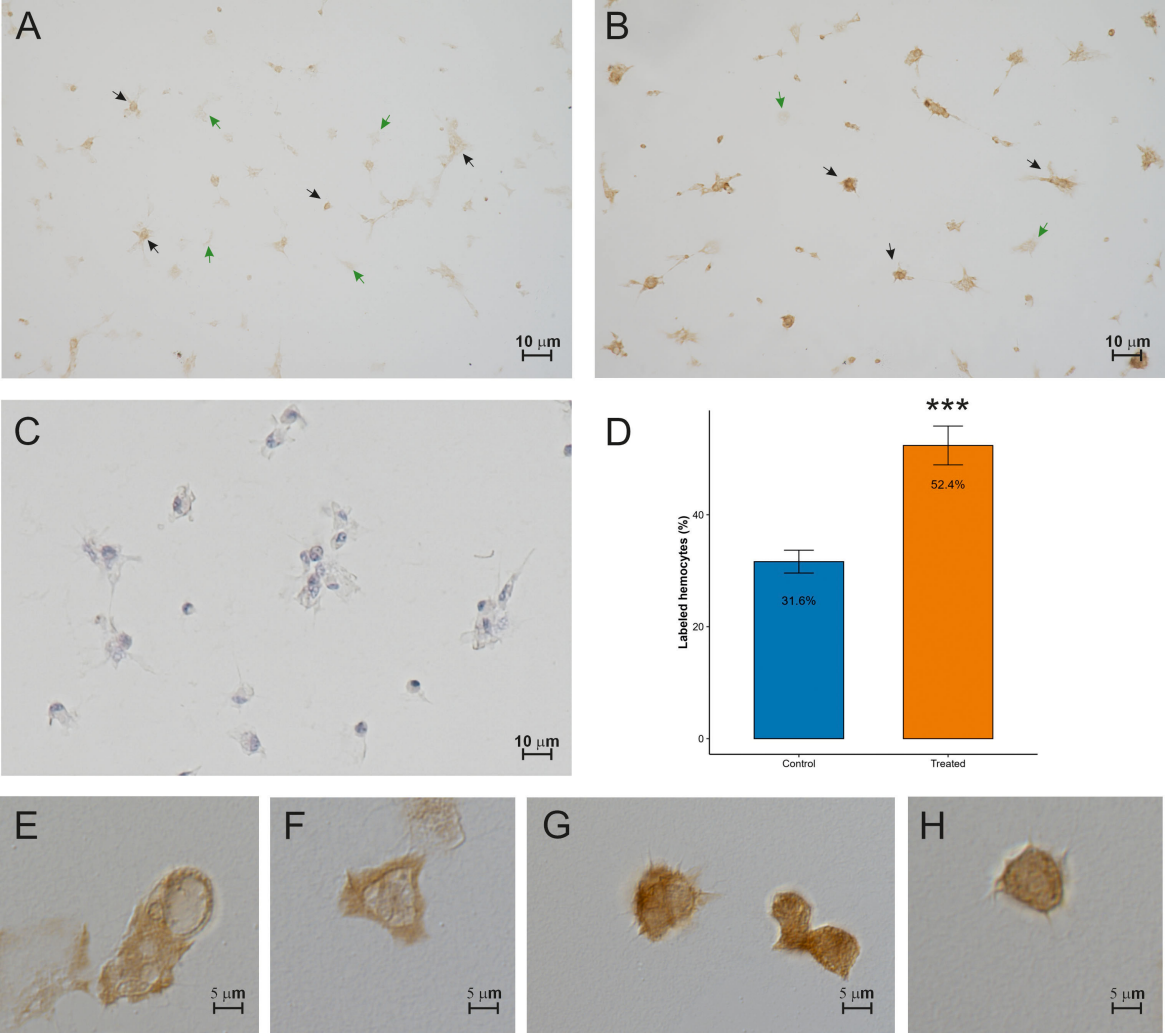
Immunocytological analysis of haemocytes stained with the anti-PcC3 antibody. **(A)** Haemocytes not treated with LPS. **(B)** Haemocytes treated with 1 mg/mL LPS for 1h. **(C)** Haemocytes stained with hematoxylin–eosin. **(D)** Percentage of PcC3-positive haemocytes in control and LPS-treated samples (Mean ± SD). **(E–H)** Representative examples of labelled haemocytes showing cytoplasmic staining **(E, F)** or membrane-associated staining **(G, H)**. *** indicates p< 0.001.

As regard the haemocytes the anti-PcC3 antibody showed a significant increase in staining from 31.6% ± 5.8% in the control group to 52.4% ± 9.8% in the LPS-treated group (p< 0.01) (**Figure 6**). It was not possible to discriminate among the haemocyte types labeled, as all cell types appeared to be involved in the staining. However, it was possible to distinguish that in some cells the labelling appeared to be cytoplasmic, whereas in others it seemed to be restricted to the cell surface membrane. (**Figures 6E–H**).

### 3.6 Phylogenetic analysis of PcC3

**Figure 7:**
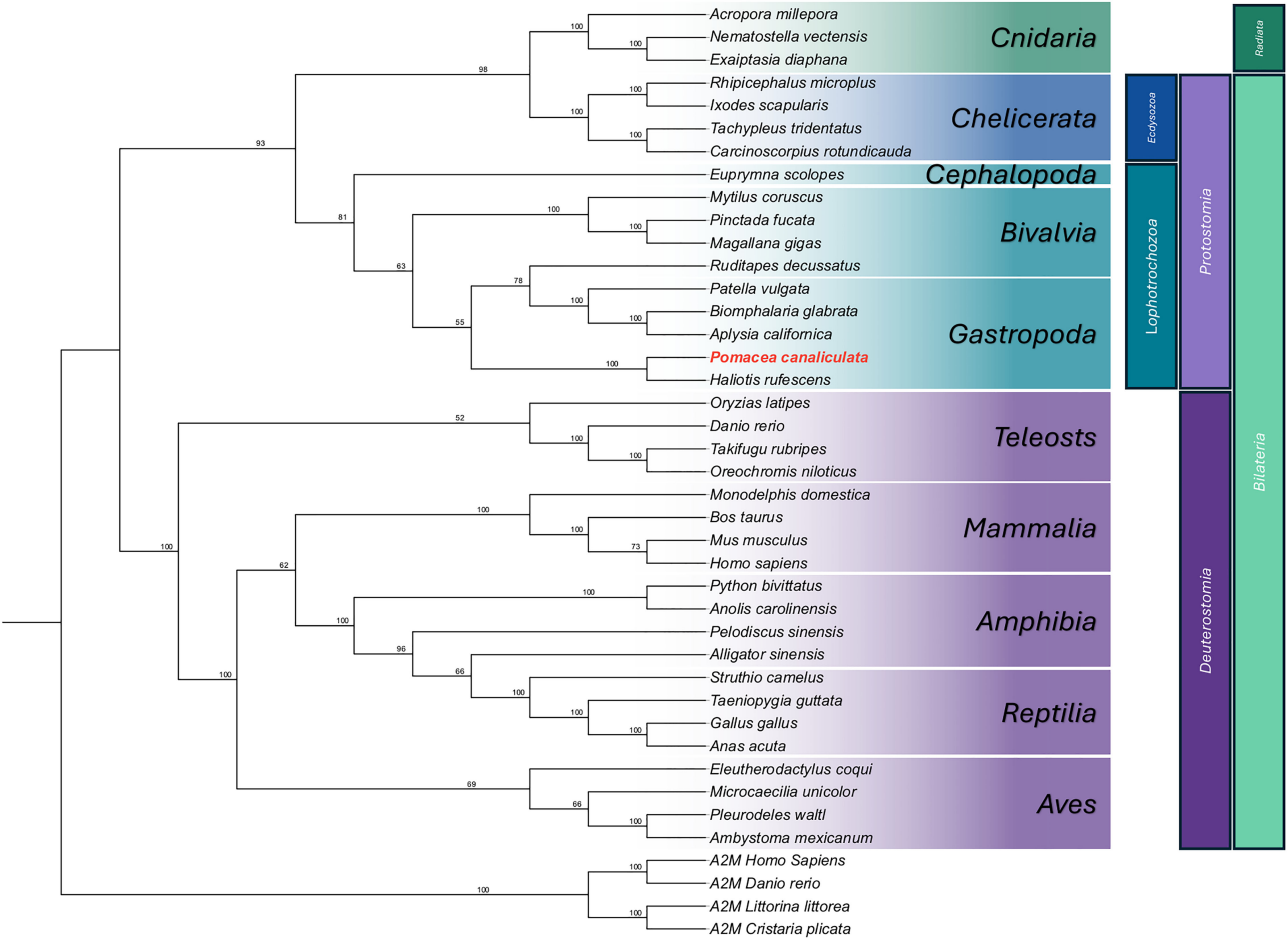
Evolutionary relationship among metazoan C3 proteins. Bootstrap confidence values indicated at the left of each branch. The tree is rooted using alpha-2 macroglobulin (H2M) sequences from multiple taxa as the outgroup. Accession numbers reported in **Supplementary Table 3**.

Phylogenetic tree constructed with the maximum likelihood method, starting from an initial tree generated with neighbour joining and rooted using H2M (α2​-macroglobulin) from different phyla. The C3 sequences are placed in positions consistent with taxonomy, showing a clear separation between Lophotrochozoa and Ecdysozoa. The division between Protostomia and Deuterostomia is also well-marked, as is the division between Radiata and Bilateria (**Figure 7**).

## 4 Discussion

The complement system relies on C3 as its central effector molecule. If complement is an ancient and versatile molecular machine, C3 represents its functional crossroads, the point at which defence, cell–cell communication and tissue remodelling converge. Exploring PcC3 therefore meant approaching the core of the system, aiming to understand not only what it does, but also when and where the animal deploys it.

The choice of organs with which to begin this investigation was far from arbitrary. Haemocytes are, in molluscs, the functional equivalent of vertebrate leukocytes: highly mobile and reactive cells capable of shifting within minutes from a quiescent morphology to an activated state. They are the first place where one would expect to observe C3 in action. The posterior kidney, in contrast, is a more enigmatic player, frequently mentioned as a potential immunological and hematopoietic organ, yet seldom explored in depth. Examining haemocytes alongside the posterior kidney allowed us to compare a well-defined effector with a putative immunological hub, providing insight into how these compartments may cooperate to maintain organismal integrity.

To this biological rationale we added an equally important technical motivation. The complement system often relies on molecular variability: alternative isoforms, modular domains, and splicing events that can alter transcript function. Short-read technologies struggle to resolve these features, particularly when isoforms diverge in complex genomic regions. For this reason, we selected Oxford Nanopore sequencing, a platform capable of reading full-length transcripts and reconstructing complete isoform architectures. This choice was not merely methodological but strategic: if Pomacea produced alternative forms of complement-related transcripts, long-read sequencing would enable us to capture them unambiguously.

The combined analysis of sequence, gene structure and protein architecture revealed that PcC3 is highly conserved relative to homologs described in other metazoans. The gene retains a complex exon organization, and the protein preserves all hallmark domains, including the A2M regions, the thioester site, and the terminal receptor-binding domains.

In addition, the C3-convertase cleavage site responsible for anaphylatoxin generation is conserved, as is a cleavage site compatible with the generation of iC3b following activation.

The presence of basic-residue motifs compatible with iC3b-type processing suggests that regulatory proteolysis of activated C3 may have deeper evolutionary roots than currently appreciated. While vertebrate Factor I is classically described to cleave after Arg (R), the corresponding PcC3 motifs involve Lys (K) in equivalent positions, which remains compatible with cleavage specificity of trypsin-like serine proteases. Nevertheless, this observation should be considered a sequence-based hypothesis: neither a Factor I ortholog nor the exact fragment pattern (iC3b/C3dg/C3d) has been experimentally demonstrated in *P. canaliculata*. Future work combining genome mining for Factor I–like proteases and cofactors, together with *in vitro* cleavage assays and mass-spectrometry mapping of C3 fragments, will be necessary to test whether a true iC3b-like regulatory pathway operates in this species. To our knowledge, this aspect has not been previously described in molluscs and suggests that mechanisms of post-activation regulation of C3b may be more evolutionarily conserved than currently appreciated.

Conversely, additional cleavage sites that in vertebrates mediate the further processing of C3b into C3dg and C3d were not clearly identifiable in PcC3. Overall, these findings support the idea that the core effector and early regulatory features of C3 are strongly conserved, whereas later stages of fragment diversification may have undergone lineage-specific modifications.

In the *Pomacea canaliculata* genome, C3 appears as a single-copy gene in chromosome 8, and no alternative splicing variants were detected. The position of PcC3 within the phylogenetic tree, fully consistent with the known evolutionary relationships among taxa, reinforces the idea that both gene structure and protein function have been strongly conserved throughout evolution. It is therefore reasonable to assume that many of the key functions of vertebrate and invertebrate C3 such as opsonization, modulation of phagocytosis, and participation in inflammatory signalling are largely maintained in *P. canaliculata*.

Within this framework, we examined whether PcC3 effectively responds to acute immune stimulation by quantifying its transcriptional expression in different organs following LPS treatment for 1h. Even though we recognize that assessing expression at 1 h represents a limitation, what we find particularly noteworthy is that the results revealed a heterogeneous pattern: haemocytes and, unexpectedly, the ampulla exhibited strong up-regulation of PcC3 after challenge, whereas the posterior kidney showed no significant variation. Up-regulation in haemocytes aligns well with their role as central effector cells of molluscan innate immunity, with increased C3 enhancing opsonic capacity and the management of pathogens and debris. More surprising is the response of the ampulla, a structure whose physiological function remains poorly defined. The induction of PcC3 suggests that this organ may play a previously underestimated role in immune responses or, at least, in signalling processes related to injury and inflammation, opening a novel line of investigation.

Conversely, the lack of PcC3 induction in the posterior kidney following immune challenge contrasts with the proposed idea that this organ is a key site of immunity or haematopoiesis. Our results instead suggest that the posterior kidney may be less involved in the acute phase of the LPS response, or may perform more basal homeostatic functions that do not require rapid C3 up-regulation. This dissociation between circulating haemocytes and posterior-kidney behaviour indicates that the immune compartments of *P. canaliculata* are functionally more specialized than previously appreciated.

Immunohistochemical analyses provide an additional layer of interpretation for both circulating haemocytes and the posterior kidney. In haemocytes, PcC3 positivity is detected in a subset of cells, a pattern coherent with their known basal c3 transcription and with the idea that these mobile phagocytes maintain a constitutive complement “priming” that can be rapidly engaged when required. The presence of C3-positive haemocytes aligns well with their central role in opsonization and debris management with no differences between male and female animals.

In the posterior kidney, PcC3 immunoreactivity is likewise restricted to a fraction of the haemocytes located within the haemocyte islets. We hypothesize that haemocytes in the posterior kidney may be present at different stages of maturation, and that only the more mature cells express detectable levels of PcC3. From this compartment, haemocytes could be released into circulation, where they may complete or rapidly achieve functional maturation. This would explain why a larger proportion of circulating haemocytes appears involved in PcC3 expression, albeit at relatively low basal levels. These observations support the interpretation that the posterior kidney may function also as a maturation niche for haemocytes rather than merely as a passive reservoir.

When combined with qPCR data, which show higher basal PcC3 transcription in the posterior kidney compared to circulating haemocytes, an interesting picture emerges. It is indeed possible that haemocytes residing within the haemocyte islets of the posterior kidney may transcribe higher levels of C3 to be released into the circulatory system, in a manner reminiscent of the complement production observed in the mammalian liver. Once these haemocytes leave their niche, they may instead assume their immunosurveillance role, becoming competent to capture activated C3 present in the haemolymph. In this scenario, the posterior kidney maintains a background transcriptional environment in which complement components are constitutively available, while the haemocyte islets provide a stable pool of phagocytic cells poised for routine tissue maintenance rather than acute immune activation.

A second interpretive axis emerges from the increasingly well-documented involvement of complement in developmental and tissue-remodelling processes. In light of our findings and a rapidly expanding body of literature, functions once labelled as “non-canonical” now appear to be integral components of complement physiology. In several vertebrate models, C3 and other complement components participate in microglia-mediated synaptic pruning and, more broadly, in the selective elimination of cells or structures deemed supernumerary during nervous-system development. At the same time, complement plays a central role in the clearance of apoptotic cells *in vivo*, with studies in murine models showing that C3 contributes to the opsonization and removal of dying cells, with critical implications for preventing autoimmunity.

Transcriptomic analysis of the earliest post-fertilization stages in *P. canaliculata* shows that PcC3 is activated remarkably early: by 3 dpf transcription is already robust, and expression reaches a stable plateau around 9 dpf. This pattern suggests that C3 acquires functional relevance during the earliest phases of development, when intense proliferation, differentiation and tissue remodelling take place. By analogy with other systems, it is plausible that PcC3 contributes to the selective removal of supernumerary, misplaced, or damaged cells, enabling fine-scale shaping of organs and circuits, likely through the activity of phagocytic haemocytes that recognize and internalize C3-opsonized targets.

In conclusion, our data show that in *Pomacea canaliculata* C3 retains the canonical functions described for complement in a broad range of invertebrates and vertebrates, participation in immune responses, opsonization, and modulation of phagocytic activity, but also suggest a crucial involvement in self-cell management throughout development. Given the phylogenetic position of *P. canaliculata* and the strong structural conservation of PcC3, it is plausible that similar roles may be widespread among gastropods and possibly across molluscs more broadly. The view of complement not only as a defender against non-self but also as a refined instrument of self-maintenance and tissue remodelling emerges clearly in this model, opening new avenues for integrated studies of immunity, development and regeneration in Lophotrochozoa.

## Data Availability

The data presented in the study are deposited in the NCBI repository, accession number PcC3: BankIt3060955, PZ124875, Transcriptomes: PRJNA1432952.

## References

[B1] Accorsi A. Bucci L. de Eguileor M. Ottaviani E. Malagoli D. (2013). Comparative analysis of circulating hemocytes of the freshwater snail Pomacea canaliculata. Fish Shellfish Immunol. 34, 1260–1268. doi: 10.1016/j.fsi.2013.02.008 23422816

[B2] Accorsi A. Ottaviani E. Malagoli D. (2014). Effects of repeated hemolymph withdrawals on the hemocyte populations and hematopoiesis in Pomacea canaliculata. Fish Shellfish Immunol. 38, 56–64. doi: 10.1016/j.fsi.2014.03.003 24636857

[B3] Accorsi A. Pardo B. Ross E. Corbin T. J. McClain M. Weaver K. . (2025). A genetically tractable non-vertebrate system to study complete camera-type eye regeneration. Nat. Commun. 16, 6698. doi: 10.1038/s41467-025-61681-6 40770180 PMC12328594

[B4] Ballarin L. Peronato A. Malagoli D. Macor P. Sacchi S. Sales G. . (2024). Evidence of a lytic pathway in an invertebrate complement system: identification of a terminal complement complex gene in a colonial tunicate and its evolutionary implications. Int. J. Mol. Sci. 25, 11995. doi: 10.3390/ijms252211995 39596065 PMC11593599

[B5] Bergamini G. Ahmad M. Cocchi M. Malagoli D. (2021). A new protocol of computer-assisted image analysis highlights the presence of hemocytes in the regenerating cephalic tentacles of adult Pomacea canaliculata. Int. J. Mol. Sci. 22, 5023. doi: 10.3390/ijms22095023 34065143 PMC8126035

[B6] Bergamini G. Sacchi S. Ferri A. Franchi N. Montanari M. Ahmad M. . (2023). Clodronate liposome-mediated phagocytic hemocyte depletion affects the regeneration of the cephalic tentacle of the invasive snail, Pomacea canaliculata. Biology 12, 992. doi: 10.3390/biology12070992 37508422 PMC10376890

[B7] Boraldi F. Lofaro F. D. Accorsi A. Ross E. Malagoli D. (2019). Toward the molecular deciphering of Pomacea canaliculata immunity: first proteomic analysis of circulating hemocytes. Proteomics 19, e1800314. doi: 10.1002/pmic.201800314 30537342

[B8] Boraldi F. Lofaro F. D. Bergamini G. Ferrari A. Malagoli D. (2021). Pomacea canaliculata ampullar proteome: A nematode-based bio-pesticide induces changes in metabolic and stress-related pathways. Biology 10, 1049. doi: 10.3390/biology10101049 34681148 PMC8533556

[B9] Chen Y. Xu K. Li J. Wang X. Ye Y. Qi P. (2018). Molecular characterization of complement component 3 (C3) in Mytilus coruscus improves our understanding of bivalve complement system. Fish Shellfish Immunol. 76, 41–47. doi: 10.1016/j.fsi.2018.02.044 29486351

[B10] Cueto J. A. Rodriguez C. Vega I. A. Castro-Vazquez A. (2015). Immune defenses of the invasive apple snail pomacea canaliculata (Caenogastropoda, Ampullariidae): phagocytic hemocytes in the circulation and the kidney. PLoS One 10, e0123964. doi: 10.1371/journal.pone.0123964 25893243 PMC4404100

[B11] Dodds A. W. (2002). Which came first, the lectin/classical pathway or the alternative pathway of complement? Immunobiology 205, 340–354. doi: 10.1078/0171-2985-00137 12395998

[B12] Elvington M. Liszewski M. K. Atkinson J. P. (2016). Evolution of the complement system: from defense of the single cell to guardian of the intravascular space. Immunol. Rev. 274, 9–15. doi: 10.1111/imr.12474 27782327 PMC5108576

[B13] Fernández-Sánchez L. Ruiz-Conca M. Estalrich M. Tebar E. Tornero L. Kutsyr O. . (2025). Study of complement system C3 protein expression in glial cells during retinal neurodegeneration. Acta Ophthalmol. 103. doi: 10.1111/aos.17396 40046247

[B14] Franchi N. Ballarin L. (2014). Preliminary characterization of complement in a colonial tunicate: C3, Bf and inhibition of C3 opsonic activity by compstatin. Dev. Comp. Immunol. 46, 430–438. doi: 10.1016/j.dci.2014.05.014 24877658

[B15] Franchi N. Loriano B. (2017). Morula cells as key hemocytes of the lectin pathway of complement activation in the colonial tunicate Botryllus schlosseri. Fish Shellfish Immunol. 63, 157–164. doi: 10.1016/j.fsi.2017.02.003 28189764

[B16] Gorbushin A. M. (2018). Immune repertoire in the transcriptome of Littorina littorea reveals new trends in lophotrochozoan proto-complement evolution. Dev. Comp. Immunol. 84, 250–263. doi: 10.1016/j.dci.2018.02.018 29501422

[B17] Guo Q. Yang W. Shan W. Yao H. Shi X. Wang L. . (2025). CTSL-2 upon specifically recognizing Vibrio splendidus directly cleaves complement C3 to promote the bacterial phagocytosis and degradation in oyster. Cell Commun. Signal. 23, 198. doi: 10.1186/s12964-025-02205-z 40275325 PMC12023428

[B18] Gurol T. Zhou W. Deng Q. (2016). MicroRNAs in neutrophils: potential next generation therapeutics for inflammatory ailments. Immunol. Rev. 273, 29–47. doi: 10.1111/imr.12450 27558326

[B19] Kristensen S. Årseth C. Ryan L. Ullmann S. Lambris J. D. Mollnes T. E. . (2025). Exploring the role of intracellular C3 in activation of the NLRP3 inflammasome in human macrophages. Immunobiology 230, 153061. doi: 10.1016/j.imbio.2025.153061 38826717

[B20] Li J. Gao J. Chu C. (2025). Identification of apple snails from other snails in snail food with quantitative PCR based on TaqMan-MGB probe. J. Food Compos. Anal. 148, 108508. doi: 10.1016/j.jfca.2025.108508 38826717

[B21] Macor P. Tedesco F. (2007). Complement as effector system in cancer immunotherapy. Immunol. Lett. 111, 6–13. doi: 10.1016/j.imlet.2007.04.014 17572509

[B22] Marino R. Kimura Y. Santis R. D. Lambris J. D. Pinto M. (2002). Complement in urochordates: cloning and characterization of two C3-like genes in the ascidian Ciona intestinalis. Immunogenetics 53, 1055–1064. doi: 10.1007/s00251-001-0421-9 11904683

[B23] Mastellos D. C. Hajishengallis G. Lambris J. D. (2024). A guide to complement biology, pathology and therapeutic opportunity. Nat. Rev. Immunol.. 24, 118–141. doi: 10.1038/s41577-023-00926-1 37670180

[B24] Meng F. Sun Y. Liu X. Wang J. Xu T. Wang R. (2012). Analysis of C3 suggests three periods of positive selection events and different evolutionary patterns between fish and mammals. PLoS One 7, e37489. doi: 10.1371/journal.pone.0037489 22624039 PMC3356312

[B25] Peng M. Niu D. Wang F. Chen Z. Li J. (2016). Complement C3 gene: Expression characterization and innate immune response in razor clam Sinonovacula constricta. Fish shellfish Immunol. 55, 223–232. doi: 10.1016/j.fsi.2016.05.024 27231190

[B26] Peronato A. Minervini G. Tabarelli M. Ballarin L. Franchi N. (2021). Characterisation and functional role of a novel C1qDC protein from a colonial ascidian. Dev. Comp. Immunol. 122, 104077. doi: 10.1016/j.dci.2021.104077 33905781

[B27] Peronato A. Drago L. Rothbächer U. Macor P. Ballarin L. Franchi N. (2020a). Complement system and phagocytosis in a colonial protochordate. Dev. Comp. Immunol. 103, 103530. doi: 10.1016/j.dci.2019.103530 31669308

[B28] Peronato A. Franchi N. Ballarin L. (2020b). Complement components as markers of hemocyte differentiation in the colonial ascidian Botryllus schlosseri. Isj-Invertebrate Survival J. 17, 25–25. Available online at: http://gateway.webofknowledge.com/gateway/Gateway.cgi?GWVersion=2&SrcAuth=mekentosj&SrcApp=Papers&DestLinkType=FullRecord&DestApp=WOS&KeyUT=000520406100004 (Accessed March 02, 2020).

[B29] Peronato A. Franchi N. Ballarin L. (2020c). Insights into the Complement System of Tunicates: C3a/C5aR of the Colonial Ascidian Botryllus schlosseri. Biol. (Basel) 9, 263. doi: 10.3390/biology9090263 32882947 PMC7565592

[B30] Pinto M. R. Chinnici C. M. Kimura Y. Melillo D. Marino R. Spruce L. A. . (2003). CiC3-1a-mediated chemotaxis in the deuterostome invertebrate Ciona intestinalis (Urochordata). J. Immunol. 171, 5521–5528. doi: 10.4049/jimmunol.171.10.5521 14607959

[B31] Pittaluga A. Torre V. Olivero G. Rosenwasser N. Taddeucci A. (2025). Non-canonical roles of complement in the CNS: from synaptic organizer to presynaptic modulator of glutamate transmission. Curr. Neuropharmacol. 23, 820–834. doi: 10.2174/011570159x327960240823065729 39817397 PMC12163473

[B32] Poole A. Z. Kitchen S. A. Weis V. M. (2016). The role of complement in Cnidarian-Dinoflagellate symbiosis and immune challenge in the sea anemone Aiptasia pallida. Front. Microbiol. 7. doi: 10.3389/fmicb.2016.00519 27148208 PMC4840205

[B33] Rahman J. Singh P. Merle N. S. Niyonzima N. Kemper C. (2020). Complement’s favourite organelle—Mitochondria? Br. J. Pharmacol. 178, 2771–2785. doi: 10.1111/bph.15238 32840864 PMC8359399

[B34] Ricklin D. Reis E. S. Lambris J. D. (2016a). Complement in disease: a defence system turning offensive. Nat. Rev. Nephrol. 12, 383–401. doi: 10.1038/nrneph.2016.70 27211870 PMC4974115

[B35] Ricklin D. Reis E. S. Mastellos D. C. Gros P. Lambris J. D. (2016b). Complement component C3 - The “Swiss Army Knife” of innate immunity and host defense. Immunol. Rev. 274, 33–58. doi: 10.1111/imr.12500 27782325 PMC5427221

[B36] Sacchi S. Malagoli D. Franchi N. (2024). The invertebrate immunocyte: A complex and versatile model for immunological, developmental, and environmental research. Cells 13, 2106. doi: 10.3390/cells13242106 39768196 PMC11674123

[B37] Sayegh E. T. Bloch O. Parsa A. T. (2014). Complement anaphylatoxins as immune regulators in cancer. Cancer Med. 3, 747–758. doi: 10.1002/cam4.241 24711204 PMC4303144

[B38] Sun J. Wang L. Song L. (2023). The primitive complement system in molluscs. Dev. Comp. Immunol. 139, 104565. doi: 10.1016/j.dci.2022.104565 36216083

[B39] Sunyer J. O. Tort L. Lambris J. D. (1997). Structural C3 diversity in fish: characterization of five forms of C3 in the diploid fish Sparus aurata. J. Immunol. 158, 2813–2821. doi: 10.4049/jimmunol.158.6.2813 9058817

[B40] Vorup-Jensen T. Jensen R. K. (2018). Structural immunology of complement receptors 3 and 4. Front. Immunol. 9. doi: 10.3389/fimmu.2018.02716 30534123 PMC6275225

[B41] Wang S. Wang R. Xu T. (2013). The evolutionary analysis on complement genes reveals that fishes C3 and C9 experience different evolutionary patterns. Fish Shellfish Immunol. 35, 2040–2045. doi: 10.1016/j.fsi.2013.10.018 24184007

[B42] Wang L. Zhang H. Wang L. Zhang D. Lv Z. Liu Z. . (2017). The RNA-seq analysis suggests a potential multi-component complement system in oyster Crassostrea gigas. Dev. Comp. Immunol. 76, 209–219. doi: 10.1016/j.dci.2017.06.009 28645512

[B43] Woo J. J. Pouget J. G. Zai C. C. Kennedy J. L. (2020). The complement system in schizophrenia: where are we now and what’s next? Mol. Psychiatr. 25, 114–130. doi: 10.1038/s41380-019-0479-0 31439935

