# Supplementary material for: C3 in Pomacea canaliculata: a conserved effector with developmental and immune roles
Source: Front Cell Infect Microbiol. 2026 Jun 30;16:1782659. doi: 10.3389/fcimb.2026.1782659 (PMC13365339; doi:10.3389/fcimb.2026.1782659)
Supplement: Supplementary file 1 [file Table1.docx]

| **Primer** | **Sequence** | **Use** |
| --- | --- | --- |
| PC3_rtF1 | ACCTTCTCACTGACCAACAAAGT | qPCR |
| PC3_rtR1 | CTAGCAAATCTCCCATGGCACTA | qPCR |
| PC3_rtF2 | AGCCAGCGAAAGTTCAAGTTTAC | qPCR |
| PC3_rtR2 | GCCAGTCTTTATCATGCCATGTG | qPCR |
| PC3_F1 | TAGTGCCATGGGAGATTTGCTAG | PCR – ISH |
| PC3_R1 | TGTACCTCTTCTCCAAGTGATGC | PCR – ISH |
| PcC3_F2 | TGTTGCAGCAATTCTTCTGG | PCR – ISH |
| PcC3_R2 | CACATACCTCCATGCACAGG | PCR – ISH |
| PcC3_F3 | CCTGTGCATGGAGGTATGTG | PCR – ISH |
| PcC3_R3 | CACCGTCAACACGTTGGTAG | PCR – ISH |

**Table 1** Primers
