# Supplementary material for: C3 in Pomacea canaliculata: a conserved effector with developmental and immune roles
Source: Front Cell Infect Microbiol. 2026 Jun 30;16:1782659. doi: 10.3389/fcimb.2026.1782659 (PMC13365339; doi:10.3389/fcimb.2026.1782659)
Supplement: Supplementary file 2 [file Table2.docx]

| **SRA ID** | **SampleName** |  |
| --- | --- | --- |
| SRR7454773 | adult female blood |  |
| SRR7454782 | adult female blood |  |
| SRR7454783 | adult female blood |  |
| SRR7454784 | adult female blood |  |
| SRR7454785 | adult male blood |  |
| SRR7454786 | adult female blood |  |
| SRR7454787 | adult female blood |  |
| SRR7454788 | adult female blood |  |
| SRR7454789 | adult female blood |  |
| SRR7454790 | adult male blood |  |
| SRR7454791 | adult male blood |  |
| SRR7454774 | adult male blood |  |
| SRR7454792 | adult female blood |  |
| SRR7454793 | adult female blood |  |
| SRR7454794 | adult female blood |  |
| SRR7454795 | adult female blood |  |
| SRR7454796 | adult male blood |  |
| SRR7454797 | adult female blood |  |
| SRR7454798 | adult female blood |  |
| SRR7454799 | adult male blood |  |
| SRR7454800 | adult female blood |  |
| SRR7454801 | adult female blood |  |
| SRR7454775 | adult male blood |  |
| SRR7454802 | adult female blood |  |
| SRR7454803 | adult male blood |  |
| SRR7454804 | adult female blood |  |
| SRR7454805 | adult male blood |  |
| SRR7454806 | adult female blood |  |
| SRR7454807 | adult female blood |  |
| SRR7454808 | adult male blood |  |
| SRR7454809 | adult female blood |  |
| SRR7454810 | adult male blood |  |
| SRR7454811 | adult male blood |  |
| SRR7454776 | adult female blood |  |
| SRR7454812 | adult female blood |  |
| SRR7454813 | adult female blood |  |
| SRR7454814 | adult male blood |  |
| SRR7454815 | adult female blood |  |
| SRR7454816 | adult female blood |  |
| SRR7454817 | adult female blood |  |
| SRR7454818 | adult female blood |  |
| SRR7454819 | adult male blood |  |
| SRR7454820 | adult female blood |  |
| SRR7454777 | adult female blood |  |
| SRR7454778 | adult male blood |  |
| SRR7454779 | adult female blood |  |
| SRR7454780 | adult female blood |  |
| SRR7454781 | adult male blood |  |
| **SRA ID** | **SampleName** | **Replicate** |
| SRR7224629 | 2dpf embryos | 1 |
| SRR7224630 | 2dpf embryos | 2 |
| SRR7224631 | 2dpf embryos | 3 |
| SRR7224632 | 3dpf embryos | 1 |
| SRR7224633 | 3dpf embryos | 2 |
| SRR7224634 | 3dpf embryos | 3 |
| SRR7224635 | 4dpf embryos | 1 |
| SRR7224636 | 4dpf embryos | 2 |
| SRR7224637 | 4dpf embryos | 3 |
| SRR7224638 | 5dpf embryos | 1 |
| SRR7224669 | 5dpf embryos | 2 |
| SRR7224670 | 5dpf embryos | 3 |
| SRR7224671 | 6dpf embryos | 1 |
| SRR7224672 | 6dpf embryos | 2 |
| SRR7224665 | 6dpf embryos | 3 |
| SRR7224666 | 7dpf embryos | 1 |
| SRR7224667 | 7dpf embryos | 2 |
| SRR7224668 | 7dpf embryos | 3 |
| SRR7224676 | 7dpf embryos | 4 |
| SRR7224677 | 9dpf embryos | 1 |
| SRR7224641 | 9dpf embryos | 2 |
| SRR7224640 | 9dpf embryos | 3 |
| SRR7224642 | 11dpf embryos | 1 |
| SRR7224645 | 11dpf embryos | 2 |
| SRR7224644 | 11dpf embryos | 3 |
| SRR7224647 | 11dpf embryos | 4 |
| SRR7224646 | 13dpf embryos | 1 |
| SRR7224649 | 13dpf embryos | 2 |
| SRR7224648 | 13dpf embryos | 3 |
| SRR7224663 | 16dpf embryos | 1 |
| SRR7224660 | 16dpf embryos | 2 |
| SRR7224661 | 16dpf embryos | 3 |
| SRR7224673 | 16dpf embryos | 4 |
| SRR7224678 | 19dpf embryos | 1 |
| SRR7224664 | 19dpf embryos | 2 |
| SRR7224639 | 19dpf embryos | 3 |
| SRR7224674 | 19dpf embryos | 4 |
|  |  |  |
| **SRA ID** | **SampleName** |  |
| SRR25525896 | GSM7681348_Hatchling_IV |  |
| SRR25525897 | GSM7681348_Hatchling_IV |  |
| SRR25525898 | GSM7681348_Hatchling_IV |  |
| SRR25525899 | GSM7681348_Hatchling_IV |  |
| SRR25525900 | GSM7681347_Hatchling_III |  |
| SRR25525901 | GSM7681347_Hatchling_III |  |
| SRR25525902 | GSM7681347_Hatchling_III |  |
| SRR25525903 | GSM7681347_Hatchling_III |  |
| SRR25525904 | GSM7681346_Hatchling_II |  |
| SRR25525905 | GSM7681346_Hatchling_II |  |
| SRR25525906 | GSM7681346_Hatchling_II |  |
| SRR25525907 | GSM7681346_Hatchling_II |  |
| SRR25525908 | GSM7681345_Hatchling_I |  |
| SRR25525909 | GSM7681345_Hatchling_I |  |
| SRR25525910 | GSM7681345_Hatchling_I |  |
| SRR25525911 | GSM7681345_Hatchling_I |  |
