# Supplementary material for: C3 in Pomacea canaliculata: a conserved effector with developmental and immune roles
Source: Front Cell Infect Microbiol. 2026 Jun 30;16:1782659. doi: 10.3389/fcimb.2026.1782659 (PMC13365339; doi:10.3389/fcimb.2026.1782659)
Supplement: Supplementary file 3 [file Table3.docx]

Tree accessions

| **Accession** | **Description** |
| --- | --- |
| ABK78771.2 | complement C3 [Acropora millepora] |
| XP_006023407.1 | complement C3 [Alligator sinensis] |
| XP_069460432.1 | complement C3 [Ambystoma mexicanum] |
| XP_068520412.1 | complement C3 [Anas acuta] |
| XP_003216795.1 | complement C3 [Anolis carolinensis] |
| XP_005093011.2 | complement C3 [Aplysia californica] |
| XP_055895877.1 | complement C3-like [Biomphalaria glabrata] |
| NP_001035559.2 | complement C3 preproprotein [Bos taurus] |
| AAQ08323.1 | complement component 3-like protein [Carcinoscorpius rotundicauda] |
| XP_009293521.1 | complement C3 [Danio rerio] |
| XP_066449838.1 | complement C3 [Eleutherodactylus coqui] |
| ACF04700.1 | complement component C3-like protein [Euprymna scolopes] |
| KXJ21803.1 | Complement C3 [Exaiptasia diaphana] |
| NP_001383072.1 | complement C3 precursor [Gallus gallus] |
| XP_048245024.1 | complement C3-like [Haliotis rufescens] |
| NP_000055.2 | complement C3 preproprotein [Homo sapiens] |
| XP_040079162.1 | complement C3 [Ixodes scapularis] |
| AHK60744.1 | complement component 3 [Magallana gigas] |
| XP_030052736.1 | complement C3 [Microcaecilia unicolor] |
| XP_003340773.2 | complement C3 [Monodelphis domestica] |
| NP_033908.2 | complement C3 preproprotein [Mus musculus] |
| AXS68445.1 | complement component 3 [Mytilus coruscus] |
| BAH22725.1 | complement component C3 precursor [Nematostella vectensis] |
| XP_005454885.3 | complement C3 [Oreochromis niloticus] |
| XP_023815099.1 | complement C3 [Oryzias latipes] |
| XP_050417000.1 | complement C3 [Patella vulgata] |
| XP_006124815.1 | complement C3 [Pelodiscus sinensis] |
| QVN30053.1 | complement component 3 [Pinctada fucata] |
| XP_069086519.1 | complement C3 [Pleurodeles waltl] |
| XP_025112875.1 | complement C3-like [Pomacea canaliculata] |
| XP_007423955.1 | complement C3 [Python bivittatus] |
| XP_037273030.1 | LOW QUALITY PROTEIN: complement C3-like [Rhipicephalus microplus] |
| ACN37845.1 | complement component C3 [Ruditapes decussatus] |
| XP_068779081.1 | complement C3 [Struthio camelus] |
| BAH02276.1 | complement component 3 [Tachypleus tridentatus] |
| XP_072776166.1 | complement C3 isoform X1 [Taeniopygia guttata] |
| XP_003972135.2 | complement C3 [Takifugu rubripes] |
| ACF39935.1 | alpha2-macroglobulin [Cristaria plicata] |
| XP_001345438.8 | alpha-2-macroglobulin-like isoform X1 [Danio rerio] |
| spP01023.3 | A2MG_HUMAN |
| AIC31934.1 | alpha-2-macroglobulin-like protein [Littorina littorea] |
